# The centrality of “family first” among Chinese migrants in determining experiences of palliative care: An integrative literature review

**DOI:** 10.1177/26323524261437364

**Published:** 2026-04-18

**Authors:** Xuan Wang, Deborah Raphael, Merryn Gott, Jackie Robinson

**Affiliations:** 1 School of Nursing, University of Auckland, New Zealand

**Keywords:** palliative care, cultural safety, health inequities, culturally competent care, transients and migrants

## Abstract

To reduce the health inequalities, it is important to understand the experiences of Chinese migrants receiving palliative care. This study focuses on how health systems can enhance the cultural safety of palliative care for Chinese migrants. How do cultural values and migration experiences shape Chinese families’ experience of palliative care? To review the evidence relating to the experiences of Chinese migrants and their families receiving palliative care. This integrative review included quantitative and qualitative studies reported in English. Study quality was assessed using the Critical Appraisal Skills Programme. Results were analyzed using thematic analysis. An electronic database search of Medline, PubMed, CINAHL, Scopus, Embase, and PsycINFO was performed. Eleven papers were included. Thematic analysis identified four themes: self-isolation, far from help, sociocultural shifts experienced by migrants, and trust problems arising from migrant experience. These themes were underpinned by a common belief of “family first.” With this core belief, patients and families make decisions that they believe will benefit the family. This review identifies the centrality of “family first” among Chinese migrants in determining experiences of palliative care. More research is needed to develop culturally appropriate palliative care services to support the growing global population of Chinese migrants.

## Introduction

In 2020, 3.6% of the global population were migrants.^1^ In this context, healthcare access and utilization for migrants have become an essential subject.^2^ Migrant populations often have unique healthcare needs, preferences, and expectations of health services.^2^ They also may face barriers to their use of health services in the host countries.^2^ For migrants, the low uptake of health services may result from previous negative experiences of healthcare in host countries, such as discrimination, administrative barriers, language barriers, or cultural barriers.^3^ These barriers are detrimental to public health and result in health inequities.

Health inequities for migrants have been recognized in palliative care services, including primary palliative care and specialist palliative care.^4,5^ Previous research reports lower utilization of palliative care services for minoritized ethnic groups.^6,7^ This inequitable access to palliative care may contribute to the higher use of acute services among migrants.^6,7^ Factors impacting access to palliative care for people from migrant groups include financial barriers, lack of congruence between the health system in their country of origin compared to their host country, cultural values which differ from those of their host country, and language barriers.^8–10^ All these factors can contribute to adverse perceptions of palliative care, difficulty building trust with health professionals, and negative experiences of the health system.^11^

Culture is an important factor influencing the delivery of palliative care. In the Asian sociocultural context, non-disclosure of truth in relation to diagnosis and prognosis is considered an acceptable practice in palliative care.^12^ In addition, a choice place of death requires balancing against the sociocultural environment in which the patient lives.^13^ Traditional cultural values held by Chinese migrants may conflict with the values of the dominant culture of the host country.^14^ This is important because cultural beliefs related to death and dying determine how palliative care services are developed. The development of palliative care has been profoundly influenced by Western religion and culture with the first hospices established by religious organizations in Dublin in 1879 ^15^ and the first modern hospice, St. Christopher’s, opening in London in 1967.^16^ Furthermore, the diffusion of the concept of palliative care has been slow across Asia. In particular, palliative care is not widely accepted by Chinese people because of the belief that palliative care involves “being abandoned of living and waiting for death.”^17^ In traditional Chinese culture, people often believed “人定胜天” (humans can triumph the heaven).^18^ This statement comes from *Records of the Grand Historian—The Biographies of Diviners*, written by Sima Qian of the Western Han dynasty (ca. 145–86 BCE). The complete sentence is “夫运筹策于帷幄之中，决胜千里之外，人定亦能胜天也○” (To devise strategies within the command tent and decide victory a thousand li (1 li is about 415 m) away—human determination, likewise, can prevail over the Mandate of Heaven.) There is evidence that this cultural value has a significant influence on the attitudes of Chinese people about the use of medical interventions in end-of-life care. For example, a cross-sectional study from China showed that 88% of participants identified life-prolonging treatment as a goal of end-of-life care.^19^

Chinese migrants have to accept the Western cultural values that underpin palliative care services in their host country when they use services. Therefore, there is increasing attention in Western countries to establish culturally safe palliative care approaches for Chinese migrants. The concept of cultural safety was developed by Māori nurse, Irihapeti Ramsden, as a means of addressing the colonial context shaping the health and wellness of Indigenous peoples in Aotearoa New Zealand.^20^ Culturally safe practice encompasses self-awareness of the healthcare provider’s own culture and an analysis of positional power that can serve to police or restrict cultural norms or values of certain groups.^21^ Cultural safety, as opposed to cultural competence, additionally encompasses an interrogation of the healthcare provider’s own biases or assumptions^22^ and can also be defined as an outcome of care as defined by the recipient and/or their family members.^23^ The concept of cultural safety is used to inform the review presented in this paper.

When looking at the experiences of Chinese migrants is also important to recognize that migration itself is a social determinant of health.^24^ However, less attention has been paid to this aspect of migrant experience, with previous research predominantly focused upon the impact of culture, rather than the impact of migration, which often includes reduced socio-economic status and diminished social network support.^25^

To reduce the inequities that arise in palliative care, it is important to explore strategies that are culturally appropriate and responsive to the needs of migrants. So, doing necessitates understanding migrants’ experiences of and need for culturally safe palliative care services.^26^ Chinese migrants are one of the largest migrant populations in the world.^27^ The aim of this review is to synthesize the available evidence related to experiences of Chinese migrants and their families receiving palliative care.

## Method

An integrative review was conducted, which focused on providing a synthesis of knowledge and evaluating the applicability of key findings to the research aim.^28^ The integrative review design was chosen to gain broad knowledge of the palliative care experience of Chinese migrants as patients or family caregivers. The integrative review was conducted following the methodological framework proposed by Whittemore and Knafl,^29^ which includes five stages: problem identification, literature search, data evaluation, data analysis, and presentation. To enhance the transparency of the search and selection process, the PRISMA flow diagram was adopted to illustrate the identification, screening, eligibility, and inclusion of studies (Supplemental Material).^30^

In line with the cultural safety framing of this paper, it is important to disclose the positionality of the authors in relation to the topic being studied, particularly in terms of ethnicity, given that the focus of the paper is on Chinese migrants. The first author, X.W., is Chinese and currently living in Aotearoa NZ and identifies as a Chinese migrant; M.G. is English and Welsh and has lived in Aotearoa NZ for 15 years and identifies as a British migrant; D.R. and J.R. are Pakeha (NZ European) New Zealanders and do not identify as migrants.

### Eligibility criteria

The inclusion criteria were determined before searching the literature^31^ (see Table 1 for details).

**Table 1.** Inclusion and exclusion criteria.

	Inclusion	Exclusion
Topics	Palliative care	Advanced care planning
Type of studies	Studies were empirical research Studies with qualitative, quantitative, and mixed methods were included	Studies were non-empirical research
Type of participants	Participants needed to be Chinese migrants aged over 18 years Participants had to have been diagnosed with a life-limiting illness or be a family member of someone diagnosed with a life-limiting illness and have experience with palliative care. This included both generalist and specialist palliative care services provided in any clinical setting Only the views of patients or their families were included, these views needed to be presented in such a way that they could be extracted and analyzed separately from other data	Participants who were not Chinese migrants or less than 18 years old were excluded Participants were healthcare providers
Type of outcomes	Studies were included if the aim and findings clearly related to the experience of palliative care and end-of-life care for Chinese migrants and their family members	Studies where the findings only related to general healthcare were excluded Studies that focused only on the views of professional health providers were excluded Studies regarding preference for palliative care for Chinese migrants were not included Quantitative studies that did not explain results with Chinese migrants separately were excluded
Type of language	English articles were included Other language articles with English translation were included	Other language articles were excluded
Type of publication	Studies published in English in peer-reviewed journals	
Published time	No limit	

Of note, articles were excluded if they focused solely on the topic of Advanced Care Planning. Advance Care Planning is a process of thinking and talking about values and goals, and eliciting preferences for current and future healthcare, which is not tied to the diagnosis of a life-limiting condition.^32,33^ As such, it is a process that is not specific to palliative care, but should be available to the general public.

### Search strategy

**Figure 1.**
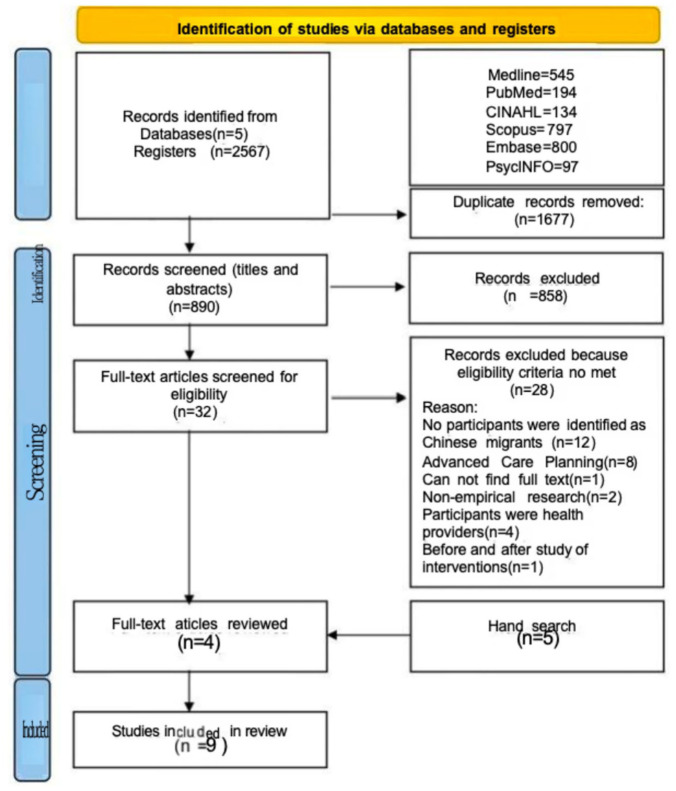
The PRISMA flow diagram.^30^

The search strategy is described in Table 2. The literature search was conducted in July 2024 and updated in 2025 (although no additional studies were identified). Before searching, a specialist subject librarian was consulted to review the search strategy. Six databases were used: Medline, PubMed, CINAHL, Scopus, Embase, and PsycINFO. Additionally, a manual search of the relevant studies’ reference lists and Google Scholar was carried out. All articles were checked for eligibility based on titles and abstracts. Those studies that met the inclusion criteria after screening the title and abstract were reviewed by full text. Studies were included if the full text met the inclusion criteria. Full included articles were screened by three independent authors. Conflicts were resolved by discussion (see Figure 1). Zotero was used to keep track of the process of selecting studies.

**Table 2.** Search strategy.

Chinese migrants	Title/abstract	“Chinese migrant” OR “Asian” OR “Chinese” OR “Chinese people” OR “Chinese emigration” OR “Chinese immigration”
		And
Palliative care; end-of-life care	Title/abstract	“Palliative care” OR “Bereavement care” OR “Terminal care” OR “End-of-life care” OR “Hospice*” OR “Supportive care”
		And
Experience	Text word	“experience” OR “perception” OR “attitude” OR “opinion” OR “quality of healthcare” OR “patient satisfaction” OR “patient-centered care”

Figure 1 shows the PRISMA flow diagram based on the guide from Moher et al.^30^ The database search revealed 2567 records, of which 1677 records were duplicates. This yielded a total of 858 records after duplications had been excluded. After reading titles and abstracts, 32 records were found to meet the inclusion criteria. After a full text review, 28 were excluded for not meeting the inclusion criteria. A further five studies were identified after a hand search of the reference lists and a Google Scholar search. A total of nine studies were included in the review.

### Data analysis

Thematic analysis was used to analyze, evaluate, and integrate research data, guided by Braun and Clarke,^34^ following Whittemore and Knafl’s advice that integrative reviews require a common analytic framework to integrate diverse forms of evidence.^29^ All data relevant to the palliative care experiences of Chinese migrants were examined by a 5-step data analysis to identify key themes.^34^ After data familiarization of all studies, initial codes were created and recorded in NVivo. During the process of creating initial codes, the results of quantitative studies were transformed from numerical data to words. This was managed by extracting key findings and noting the direction of effect (increase, decrease, or no change), effect size or strength (small, moderate, large), statistical significance (*p* value or confidence interval), and relevant context (population, setting, measurement type). These data were then compared to, and integrated with, the initial codes, and final categories and codes were developed.^35^ The data extraction and analysis processes were reviewed by all authors. Different opinions were discussed within the research team, and a consensus was reached.

## Results

The studies included in this review present a diversity of evidence across different study designs, countries, and healthcare settings. Table 3 describes the key characteristics of the included studies. Nine articles were identified from eight different studies^36–44^ with two articles reporting data from the same study.^41,42^ Two were master’s theses.^43,44^ Eight studies were situated across four countries: the United States, Canada,^38–42,44^ Australia,^37^ and New Zealand.^43^ Six studies were qualitative,^37,38,41–44^ and three were quantitative.^36,39,40^ Most studies explored all types of palliative care delivery^36,38–40,43,44^ and three were focused just on community palliative care.^37,41,42^

**Table 3.** Summary of included articles.

Author	Year	Country	Aim	Participants (age)	Setting	Method/design	Interview language	Summary of relevant findings
Quantitative								
Ngo-Metzger et al.^36^	2008	USA	To compare hospice use in the last year of life for people aged >65 years who died from cancer with a focus on Pacific Island people and Chinese Americans	184,081 patients with cancer who were non-Hispanic whites or Asian American and Pacific Islanders (AAPI) include 2145 Chinese people (aged 65 and older)	Hospice	Quantitative/A secondary analysis of routinely collected health data	*	1. For all ethnic groups, AAPIs had lower rates of hospice enrollment 2. Of those who enrolled in hospice, Chinese Americans had a similar median length of stay (26 days) compared to non-Hispanic white people
Jia et al.^39^	2024	Canada	To compare palliative care use and delivery between ethnically Chinese and non-Chinese adults in their last year of life	541,287 adults include 13,587 Chinese decedents (aged >18 years)	Clinic, home, hospital, subacute care such as post-acute rehabilitation care facility, virtual, case management, multiple settings	Quantitative/A secondary analysis of routinely collected health data	*	1. Chinese ethnicity was associated with a higher likelihood of receiving specialist and mixed models of palliative care than non-Chinese ethnicity 2. Ethnically, Chinese patients were more likely to have palliative care initiated in the last month of life, in the hospital setting, and by specialist palliative care practitioners compared to non-Chinese patients
Yarnell et al.^40^	2020	Canada	To compare end-of-life care delivered to people of Chinese and South Asian ethnicity compared with that delivered to others from the general population	967,339 decedents include 18,959 Chinese ethnicity (median age is 80; interquartile range aged 68–87)	Palliative care physician visits, ICU admissions, hospital admissions, and procedures during the final 6 months of life	Quantitative/A secondary analysis of routinely collected health data	*	1. Among decedents in Ontario, those of Chinese ethnicity were more likely to receive aggressive care and to die in intensive care than those from the general population
Qualitative								
Family members perspective								
Heidenreich et al.^37^	2014	Australia	To explore the influence of Chinese cultural norms and immigration on the experience of immigrant women of Chinese ancestry caring for a terminally ill family member at home in Sydney	Five Chinese women carers (aged 50–65)	Palliative home care	Qualitative/semistructured face-to-face, in-depth interviews	English (one of the participants needed Cantonese-speaking interpreters)	1. Chinese migration can receive limited support 2. They feel isolated due to the loss of familiar values 3. Patients and their families have emotional, physical, and financial constraints between them 4. Families have emotional, physical, and mental distress
Hathaway^43^	2009	New Zealand	To explore and describe the experiences of Chinese immigrants receiving hospice services in New Zealand	8 bereaved family members	Hospices, hospitals, or residential care facilities	Qualitative/semistructured face-to-face, in-depth interviews	English (Mandarin or Cantonese interpreter)	1. A lack of knowledge of hospice services and insufficient access to information 2. Families reported concerns about the quality of palliative home care, and they refused to enter the inpatient unit admission due to problems such as language barriers and family separation 3. Chinese migrants had a limited understanding of hospice 4. Language difficulties made participants and patients spend time searching for information and missing potential services 5. The Inpatient Unit was viewed as an appropriate place of death 6. Comparisons with care in other countries
Wu^44^	2015	Canada	To understand the lived experiences of Chinese family caregivers who have provided palliative and end-of-life care for their loved ones	Seven Chinese bereaved family caregivers (aged 50–85)	*	Qualitative/semistructured face-to-face, in-depth interviews	Chinese	1. Devoting themselves fully to caring for their loved ones 2. There is a conflict between who should disclose the illness and the lack of medical reporting 3. “Fallen Leaf Returns to the Root”* 4. The role of a family caregiver is to fulfill their familial responsibilities 5. Patients did not want to be a burden to the family 6. Tend to make comparisons between health systems
Patients’ and family members’ perspectives								
Seto Nielsen et al.^41^	2013	Canada	To describe and examine how meanings of home condition negotiations of care for Chinese immigrants with advanced cancer receiving palliative home care in Toronto, Canada	4 care recipients (aged 50–80) and 5 family caregivers	Palliative home care	Qualitative/semistructured face-to-face, in-depth interviews	English (Cantonese interpreter)	1. Overcome barriers like limited English, Chinese families can re-enforce existing transnational linkages (Chinese doctors) to receive knowledge 2. Patients identified themselves and were being re-positioned as selfish if they asserted their needs or wishes 3. The precarious and demanding nature of employment for many immigrants (and non-immigrants) meant that caregivers might be unaware of available resources
Seto Nielsen et al.^42^	2015	Canada	To describe the discursive tensions, present in-home care policies when providing palliative home care to Chinese immigrants	4 care recipients (aged 50–80) and 5 family caregivers	Palliative home care	Qualitative/semistructured face-to-face, in-depth interviews	English (Cantonese interpreter)	1. Not dying at home is not only just a cultural belief but also a choice based on multiple and conflicting considerations 2. The harmonization** of cultural differences can be better described by care recipients and caregivers than by healthcare practitioners who have difficulty in describing cultural differences. Care recipients and families were resilient in addressing language barriers 3. They often developed sophisticated approaches to work around language barriers
Patient’s perspective								
Leung et al.^38^	2024	Canada	To explore the experiences of older Chinese cancer patients to improve culturally sensitive cancer care	Twenty patients (aged 60–84)	Gastrointestinal cancer clinic	Qualitative/semistructured face-to-face, in-depth interviews	Cantonese and Mandarin	1. Acceptance and relinquishment 2. Family first 3. Self-sufficiency 4. Sociocultural barriers to supportive care

* A Chinese traditional belief, it means “a person should die in their birthplace.”

** Harmonization: Taken from the Confucian idea of “Harmony and Difference,” the idea here is to find a balance through dialogue and understanding.

### Study characteristics

Three studies used a secondary data analysis of routinely collected national health data.^36,39,40^ The main outcomes explored in these studies were health service use in the last year of life and location of death. Decedents in two of the three studies were adults (over 18 years old),^36,39^ and one study did not have any age limitations.^40^

In the qualitative studies, participants consisted of patients or family members, with sample sizes ranging from 4 to 20. One study looked at patients’ views on primary palliative care services.^37^ Two articles from one study looked at the experiences of community palliative care from four patients and five family members.^41,42^ The remaining studies focused on the families’ perspectives of different types of palliative care.^39,43,44^ Family members in three studies comprised the bereaved family.^39,43,44^ Most participants were spouses, and a small number were adult children.

Using the concept of cultural safety as a framework for this review prompted attention to be paid to whether culturally safe research methods were used and if researchers disclosed their own positionality in relation to the topic. Almost all qualitative studies used Chinese (Mandarin or Cantonese) as the language of choice for interviewing participants,^38,41–44^ with only one study using English as the interview language.^37^ Of the five remaining qualitative studies, three studies used interpreters to translate during interviews^41–43^; two studies were translated by authors who are Chinese-English bilinguals,^38,44^ with interviews conducted in Chinese. None of the authors in any of the studies stated their cultural backgrounds. In one qualitative study^44^ and one quantitative study,^39^ it appeared that the author had a Chinese cultural background because of the author’s name, although the limitations of the use of names to represent ethnicity need to be acknowledged.

### Quality appraisal results

The Critical Appraisal Skills Programme (CASP) qualitative checklist tool evaluates the quality of both quantitative and qualitative health and social care-related studies.^45,46^ Although the choice of language to be used during an interview is not a mandatory evaluation criterion of CASP, we added this category because undertaking interviews and providing questionnaires in a language preferred by the participant is a culturally safe research practice and will affect the quality of communication.^47^ Based on the CASP checklist, all studies were appraised as high quality (see Table 1).

### Overview of findings

The articles identified by the review described the experience of receiving palliative care services from the perspectives of Chinese migrant patients and their families. While positive experiences were reported in the included studies, they mainly highlighted barriers, which may reflect a prevailing research orientation toward problem identification rather than identifying mitigating and supportive factors. The review is therefore predominantly focused on those barriers that can make the use of palliative care by Chinese migrants difficult. The review identified four interconnected key themes to describe those barriers: self-isolation, far from help, sociocultural shifts experienced by migrants, and trust problems arising from migrant experience. In order to foreground migrant experiences, we have included a quotation from one of the included studies to introduce each section. To integrate the four key themes, a Chinese migrant model for palliative care was created, which synthesized all current information covered by the existing research regarding the impact of the cultural attributes and migration characteristics of Chinese migrants upon their experience of palliative care. This is presented at the end of the findings section.

### Self-isolation

I cannot ask my son to give up his job to come back to help . . . [I have] friends . . . but they all have to work and they have families and you cannot expect them to be with you all the time. (Female, family member)^37^

The first theme identified was “self-isolation,” found in four papers.^37,38,43,44^ Self-isolation describes how Chinese migrants who were receiving palliative care often refused to express their needs or join in social activities. Patients believed that asking for help was a selfish behavior and could “cause trouble” for families and friends.^37,38^ Hence, patients choose to isolate themselves by attempting to solve their problems alone. The underlying reason to self-isolate was “not to be a burden.” Often, this belief was reported by patients, however studies also found evidence that spouses of patients held a similar perspective.^44^ There was a difference, though, that the spouse thought they and their partner were a team working together to solve the problems inside the team.^44^ As a result, the couple would isolate themselves together in the process of palliative care as they did not want their children to spend money and energy on them. The couple describes being protective of their children’s heavy workload and financial burden.^38,43,44^

### Far from help

There are other family members. Now I’m only caring myself. I have got my family members in Hong Kong. (Female, family member)^37^

Five studies highlighted how family members of people with palliative care needs struggled to get the help they needed.^37,41,42,44^ This was particularly evident for first-generation migrants, where parents had also migrated to live with their children.^41,42,44^ There were three main reasons to explain what they experienced in the process of seeking help. Firstly, the family felt they had lost control of their life. Family members who could speak English had to play multiple roles in supporting the patient, including translator, manager, and health caregiver. They also had additional roles in their own life, including being a parent, employee, and spouse.^37,41,42,44^ Family members expressed a strong sense of anxiety about the possibility of the loss of their ill family member.^37,43,44^ They had trouble extricating themselves from this out-of-control state, both physically and psychologically.^37,43,44^ The second reason was related to the family’s immigration status. Apart from core family members, many family relatives and close friends were still in China.^37^ Chinese migrants found it hard to receive the support they needed from family relatives or friends who were in China. The third reason related to insufficient access to information in a language and format they could understand which also formed a barrier to Chinese migrants from receiving support.^43^ Furthermore, Chinese migrants using English as a second language found it hard to express themselves and seek medical assistance.^44^ In addition, the lack of direct access to information in English caused Chinese migrants to misunderstand and refuse some health services. This situation was worse when the translation was not accurate.^43^

### Sociocultural shifts experienced by migrants

I remember that hospice say “you should go to the hospice because you drag your wife and family down.” If I interpreted this for my father, what my father feels? I refuse . . . a lot of sentence I did not translate straight. (Male, family member)^43^

Due to sociocultural shifts resulting from migration, Chinese migrants experienced a range of difficulties, both as patients and family members. Three subthemes related to sociocultural shifts experienced by migrants were identified: the impact of the host country’s culture on individual cultural beliefs, language barriers, and culturally conflicting beliefs about death.

The host country’s culture shapes individual beliefs, and this transformation often leads to conflicts between first- and second-generation migrants when people are receiving palliative care.^1^ First-generation migrants described their concerns about differences in expectations of the kind of healthcare they will receive with their children who were growing up in host countries, which were rooted in different cultural beliefs.^43^ For example, older migrants worried that their children would prefer that they were cared for in a residential care facility rather than at home due to differences in cultural understanding regarding family care.^37^ In addition, many patients and family members expressed conflicts between Chinese migrants and their healthcare providers, especially when addressing things like the use of Chinese medicine, decision-making, and truth-telling.^43,44^ The main reason for these conflicts was the desire of Chinese migrant patients to maintain their Chinese cultural values in the context of palliative care services, often resulting in cultural needs that could not be met. Conflict was also observed due to limited experience and knowledge of palliative care, reflecting the inherent gap in understanding between patients and healthcare professionals.^44^

Problems with language barriers were also highlighted in this review. Some studies reported how Chinese migrants would feel pressure and fear in healthcare spaces where English was being spoken.^43^ However, strategies adopted by family members to overcome language barriers were identified. For example, families tried to use pictures or self-made translated word lists to support patients accessing health services.^42^ Although patients had language help from their families, difficulties communicating in English prevented them from seeking medical services.^43,44^

Death beliefs of Chinese migrants were also different from the cultural norms of Western countries. Some studies reported how Chinese migrants expected to die in a hospital. This was related to the migrants’ experiences in China, where patients received hospital-based treatment in a hospital and would often spend their last days in the hospital.^43,44^ Chinese migrants had no experience or knowledge about the process of dying in their host country or what to do after their family member had died. Hence, they preferred to stay in a hospital or hospice in the last days of life so they could seek information from healthcare professionals more readily. This finding was also reported in quantitative studies where Chinese migrants were more likely to die in the hospital.^36,39,40^ In addition, preference for place of death was consistent with the established experience of Chinese migrants using the hospital system to assist them in care after death.^43,44^

### Trust problems arising from the migrant experience

I worked hard and became very tired at night, but I still had to care for her . . . I had to care for her. You know, if the family member is not around, how can you trust the nurses? It makes a huge difference when a family member is present or not. Families are different, no matter how busy you are, you have to come. (Female, family member)^44^

There were various reasons for experiencing distrust in palliative care identified in the review. Distrust of palliative care occurred within the context of distrust for the healthcare system of their host country in general. Having to navigate an unfamiliar health system caused patients and family members anxiety.^38,41,43,44^ This was particularly acute for palliative care because patients’ and family members’ fear of death amplified their sense of unease as migrants and their discomfort with cultural differences.

Chinese migrants tended to seek health information in Chinese in order to overcome their knowledge gap about palliative care.^43^ As it was difficult for them to learn about and understand palliative care directly by themselves in English, they often chose to get advice by contacting doctors or friends in China.^41^ However, differences in the healthcare systems, misinformation about palliative care from China, conflicts between Chinese traditional beliefs about death and dying, and working with healthcare providers resulted in more confusion.^41,43,44^ In addition, expectations of healthcare in the host countries based on their previous experience in China contributed to Chinese migrants’ mistrust of the palliative care service if the service did not meet their expectations.^38,41,43,44^

#### Family first

**Figure 2.**
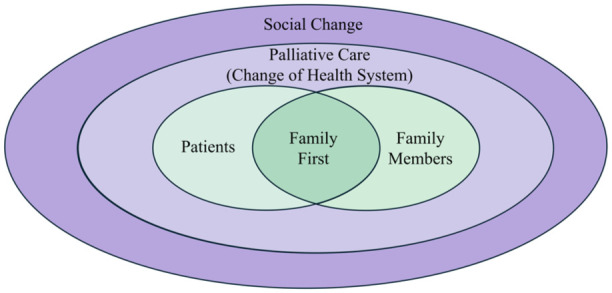
A Chinese migrant model “family first” for palliative care.

In synthesizing the evidence, four key themes were identified as presented above. Further analysis led to the realization that all themes are connected to the principle of “family first.” This can be understood as the product of both Chinese cultural values and the migration experience. To make this relationship explicit, a conceptual model rooted in Confucian ethics was developed (Figure 2). The green section of the figure derives from the themes of self-isolation and being far from help, illustrating the interconnectedness and shared cultural values between patients and their families. The purple section stems from the themes of sociocultural shifts experienced by migrants and trust problems arising from the migrant experience, highlighting the challenges brought about by the migrant experience.

## Discussion

The results from this review have highlighted how Chinese migrants experience barriers to receiving palliative care. These barriers arise as a result of both cultural and migratory influences. As a result, patients and their family members face considerable challenges in reconciling personal values and beliefs about death and dying with Western palliative care. That most research to date has focused almost exclusively on these barriers is also an important finding. Future research that adopts an appreciative inquiry approach to focus on enablers of a good palliative care experience for Chinese migrants is needed.

### Cultural influence

A key finding from this review was how patients and their partners used self-isolation as a way of protecting their family from the burden of caring for them. Feeling like a burden to others is a common concern for patients receiving palliative care.^48,49^ This concern is referred to as the care recipient’s “self-perceived burden (SPB).” SPB is a complex concept with psychological, relational, and dimensional attributes that are shaped by culture and sociopolitical structures.^50^ A systemic review found that SPB is an important issue, reported by 19%–65% of people with a life-limiting illness.^51^ However, the wider literature conceptualizes SPB at a patient level. This review highlighted by contrast how Chinese migrants’ partners seemed to share this SPB and the ways they experienced and managed this as a small unit.

Findings from this review also revealed how patients’ and, if they were married, their spouses’ self-isolation was an active response to SPB. Patients tended to place a distance from their family members by reducing connections and attempting to maintain independence.^52,53^ However, there are no specific studies that clearly identify the reasons for the behavior of patients sharing SPB with their partners and actively segregating themselves from their extended family. This is despite the fact that a Chinese study found that SPB was significantly associated with carer burden, and both were associated with levels of anxiety and depression in patients and spouses.^54^ This may explain the burden for Chinese migrants’ partners, but it still does not explain why Chinese migrants’ partners see the couple as a unit and choose self-isolating behaviors together. A further explanation may relate to the strong belief of “family first” identified in the review. This leads couples to prioritize family over other needs, even if this means being socially isolated from their wider family. The added impact of migrant status in the host country is also relevant. A lack of knowledge about palliative care, the inability to access information in their first language, and an unfamiliar health system may leave the couple feeling that they have no choice but to self-isolate. However, more research is needed to explore this in more detail.

Another related finding from this review was how Chinese families of people receiving palliative care generally reported being “far from help.” This resulted in a sense of losing control of their lives, with little support from the community and wider society. Family members are important sources of physical, mental, and financial support and provide a bridge to connect health professionals and patients.^55^ This role for the family can be associated with a significant burden.^55,56^ The review identified the importance of responding to the needs of family and recognizing the added difficulty of their role in filling service gaps in health and palliative care provision. This included acting as interpreters as well as being cultural care providers.^57^ This finding has also been reported in a study on Latino migrants, where families needed more support than non-migrants because of language and cultural barriers to accessing and navigating palliative care.^58^

### Migrant influence

The findings in this review highlight how Chinese migrants receiving palliative care face additional challenges, such as reconciling different cultural beliefs about death. Although these difficulties are identified and reported elsewhere,^42,59,60^ previous commentary has not focused on the role of sociocultural shifts related to migrant status.

Cultural transmission is the process of transferring cultural values, and can shape an individual’s response to their socio-economic and cultural environment.^61^ This process can involve vertical, oblique, or horizontal transmission.^62^ Most people experience vertical transmission within their families, however this can be disrupted by migration. Direct vertical transmission takes place between parents and their children. When it comes from peers, who are typically members of the same generation, horizontal transmission frequently occurs, particularly among young people. A third form of cultural transmission is oblique transmission that is in situations such as religious services (pastor to congregation), with political officials (politician to constituents), or in educational settings (teacher to student).^63^ The review confirms that Chinese migrants are often lack relevant knowledge and education about the health system, especially palliative care. For Chinese first-generation migrants, vertically transmitted knowledge from the country of origin proves less effective in the host society, where information is primarily acquired through oblique and horizontal transmission. Chinese migrants are missing out on potential opportunities to access palliative care, which increases health inequalities because of the absence of cultural transmission. However, this health inequality is typically not discussed by patients.

This review identified that Chinese migrants struggle to fully trust and understand the healthcare system in their host country, which provides a barrier to healthcare utilization. However, the impact of migration diminishes with each generation of migrants. First-generation migrants often face potential health inequalities due to challenges arising from migration, such as language barriers and unfamiliarity with healthcare systems. Second-generation migrants can mitigate these challenges by having access to additional information, for example from their peers. The review findings highlight that both migrations’ status and specific Chinese cultural beliefs often make the existing palliative care system inadequate to meet their needs.^64^ Three solutions can be considered in relation to these challenges. Firstly, health systems need to create more specific models of palliative care in order to respect migrant cultural beliefs and minimize health inequality. Secondly, health systems need to strengthen appropriate palliative care education in migrant communities forging relationships with these communities, which includes building trust.^58^ Finally, practicing cultural safety is a significant skill for healthcare professionals to develop in order to minimize the impact of their own biases on patients’ experiences.

The experience of migration gives rise to a general lack of trust in the host country’s healthcare system among Chinese migrants. Generally, people’s trust comes from two sources: one is based on previous experience, and the other is believing in an individual or a system.^65^ Although the importance of building trust is recognized in palliative care, the process of establishing trust with migrants may be more complex. Evidence has shown that past healthcare experience (particularly in another country’s health system) can broaden or limit a person’s range of expectations in relation to current care.^66^ When expectations are unmet, negative emotions are transformed into skepticism and distrust toward the healthcare system. In addition, Chinese migrants can easily obtain Chinese information from China.^67^ Where health services in the host country are different, and/or do not meet a person’s expectations, migrants are more likely to lose trust.

### Family-first model

Analyses concluded that the four themes identified in the review were related to the central concept of “family first.” This concept is rooted in traditional Chinese culture, and specifically Confucian family ethics, and encompasses the Confucian values of filial piety and humanity. Compared to the second-order “individual-society” model prevalent in Western culture,^45^ traditional Chinese society operates on a third-order “individual-family-society” model.^68^ Confucian family culture has shaped the health practices of Chinese migrants. Firstly, children bear the responsibility of caring for their older relatives, a duty particularly emphasized as filial piety.^69^ Secondly, family ethics play a significant role. In Confucian culture, family is not merely defined by blood ties but by ethical relationships established through filial piety (xiao), righteousness (yi), and ritual propriety (li). Family members such as stepchildren or extended relatives can be considered family members when they participate in these moral obligations and relational duties.^70^ Family members are reluctant to remain passive observers, preferring instead to participate actively, hoping their advocacy and support may contribute to the patient’s plan of treatment.^69^

Through migration, Chinese migrants strengthen and internalize the “family first” belief within their households, using it as a key strategy to manage the social and cultural challenges of migration. Historically, in Europe, the influence of the clan system and the importance of lineage in family decision-making have been broken by the Church, which has led to increased individual autonomy.^47^ Meanwhile, in China, the family system is more firmly based on Confucian family ethics, so in some cases, the family plays a crucial role in decisions.^47^ Of note, Confucian family ethics continue to be passed on even when Chinese migrants reside in the host country. Furthermore, the concept of the family is reinforced by the migrant status.^48^ However, the review also found that, for second-generation migrants, migrant status is often attenuated or even erased due to being born in the host country. As a result, those who no longer embody distinct migrant characteristics may not share a strong identification with the notion of “family first.” How this impacts experiences within a palliative care context requires further research attention.

As a foundation to such research, it is important to clarify that “family first” is not the same as “family-centered decision-making.” While family-centered decision-making is most often applied in pediatrics and intensive care settings facilitated by healthcare providers, “family first” in Chinese culture is a pervasive value spontaneously enacted by patients and their families.^49,50^ Ultimately, family first can be understood in four dimensions: (1) cultural foundation, where Confucian values position the patient as an extension of the family and emphasize communication with relatives before decisions are made; (2) emotional stress, as families bear long-term psychological and caregiving burdens, requiring time for adequate communication; (3) ethical balance, where decision-making is embedded in an emotional community shaped by shared values and responsibilities rather than motivated by preserving individual autonomy alone; and (4) treatment realities, as family resources and acceptance directly affect patients’ choices, quality of life, and the feasibility of different care options.

By emphasizing the central role of the family for Chinese migrants, the “family-first” model may enable health professionals to provide culturally safe, family-oriented palliative care. This approach respects the person’s traditions, beliefs, and emotional needs. In the practice context, “family first” may be operationalized through multidisciplinary care meetings involving family, culturally specific communication training for clinicians, and the inclusion of family-centered assessment tools. These practices not only align with patient-centered care principles but could also ensure cultural safety is maintained throughout the palliative care process. However, more research is needed to examine the value of this model in practice and support its integration into the health system.

### Methodological issues

While the reviewed studies were of high quality overall, it is important to highlight two important considerations for future research in this area. Firstly, the quality of an interview or completed survey is affected if migrants communicate in their second language,^47^ particularly in relation to the ability to express feelings and opinions.^71^ For example, a study about language use by people who emigrated as children shows that people have different self-perceptions and emotions when they are speaking a different language.^72^ In addition, differences between interpreters’ styles and translation preferences can negatively influence data quality.^73^ Only three of the included studies discussed these language issues and it’s important that future studies do so.

It is also important to recognize that no study disclosed the positionality of authors in relation to their ethnic and cultural background. This is a significant omission because all aspects of a research study are influenced by the authors’ understanding and thinking. Positionality statements are particularly important in studies where equity is a key consideration and are recommended for future research in this area.^74^

### Implications

This review has identified that the challenges experienced by Chinese migrants related to palliative care must be considered in relation to both the cultural values and migrant status of Chinese migrants. The family-first model provides guidance for clinicians and policymakers as to how to identify and meet the palliative care needs of Chinese migrants and their families.

### Strengths and limitations

The study has several strengths. From a methodological consideration, an integrative review combining the results of qualitative and quantitative analyses can lead to more comprehensive knowledge.^29^ Close cooperation and active discussion among the research team promoted the rigor of the review. Additionally, this study adds to the existing evidence by highlighting how integrating a “family-first” model into the health system may enhance cultural safety in palliative care. Incorporating family-inclusive, culturally safe practices may bridge cultural gaps and improve the quality of palliative care for Chinese patients.

However, there are some limitations to this review which relate to the quality of the included studies. As this review is a study about cultural influences, it is problematic that the authors do not disclose their positionality. Cultural and educational background may also affect one’s understanding and interpretation of the influence of culture on Chinese migrants in palliative care.

## Conclusion

Chinese migrants’ experiences of palliative care were shaped by the following four issues: self-isolation, far from help, sociocultural shifts experienced by migrants, and trust problems arising from migrant experience. The “family-first” concept helps explain the palliative care experience of Chinese migrants. More research is needed to develop culturally appropriate palliative care services to support the growing global population of Chinese migrants.

## Supplemental Material

sj-docx-1-pcr-10.1177_26323524261437364 – Supplemental material for The centrality of “family first” among Chinese migrants in determining experiences of palliative care: An integrative literature review Supplemental material, sj-docx-1-pcr-10.1177_26323524261437364 for The centrality of “family first” among Chinese migrants in determining experiences of palliative care: An integrative literature review by Xuan Wang, Deborah Raphael, Merryn Gott and Jackie Robinson in Palliative Care and Social Practice

sj-docx-2-pcr-10.1177_26323524261437364 – Supplemental material for The centrality of “family first” among Chinese migrants in determining experiences of palliative care: An integrative literature review Supplemental material, sj-docx-2-pcr-10.1177_26323524261437364 for The centrality of “family first” among Chinese migrants in determining experiences of palliative care: An integrative literature review by Xuan Wang, Deborah Raphael, Merryn Gott and Jackie Robinson in Palliative Care and Social Practice
