# Supplementary material for: The centrality of “family first” among Chinese migrants in determining experiences of palliative care: An integrative literature review
Source: Palliat Care Soc Pract. 2026 Apr 18;20:26323524261437364. doi: 10.1177/26323524261437364 (PMC13100415; doi:10.1177/26323524261437364)
Supplement: sj-docx-1-pcr-10.1177_26323524261437364 – Supplemental material for The centrality of “family first” among Chinese migrants in determining experiences of palliative care: An integrative literature review [file sj-docx-1-pcr-10.1177_26323524261437364.docx]

Supplementary Table 1

| Author (year) | year | 1 | 2 | 3 | 4 | 5 | 6 | 7 | 8 | 9 | 10 | 11 |
| --- | --- | --- | --- | --- | --- | --- | --- | --- | --- | --- | --- | --- |
| Ngo-Metzger et al. | 2008 | Yes | Yes | Yes | Yes | Yes | Yes | Yes | yes | Yes | Yes | Yes |
| Jia et al. | 2024 | Yes | Yes | Yes | Yes | Yes | Yes | Yes | Yes | Yes | Yes | Yes |
| Yarnell et al. | 2020 | Yes | Yes | Yes | Yes | Yes | Yes | Yes | Yes | Yes | Yes | Yes |
| Heidenreich et al. | 2014 | Yes | Yes | Yes | Yes | Yes | Can't tell | Yes | Yes | Yes | Yes |  |
| Hathaway. | 2009 | Yes | Yes | Yes | Yes | Yes | Yes | Yes | Yes | Yes | Yes |  |
| Wu. | 2015 | Yes | Yes | Yes | Yes | Yes | Yes | Yes | Yes | Yes | Yes |  |
| Seto Nielsen et. al. | 2013 | Yes | Yes | Yes | Yes | Yes | Yes | Yes | Yes | Yes | Yes |  |
| Seto Nielsen et. al. | 2015 | Yes | Yes | Yes | Yes | Yes | Yes | Yes | Yes | Yes | Yes |  |
| Leung. et. al. | 2024 | Yes | Yes | Yes | Yes | Yes | Yes | Yes | Yes | Yes | Yes |  |
